# Premarital Genetic Diagnosis Revealed Co-heredity Nature of Beta Globin Gene 25-26 del AA and 3’UTR+101 G-C Variants in Two Beta Thalassemia Heterozygotes

**DOI:** 10.4274/tjh.2016.0069

**Published:** 2017-03-01

**Authors:** Kanay Yararbaş, Yasemin Ardıçoğlu, Nejat Akar

**Affiliations:** 1 Düzen Laboratories Group, Ankara, Turkey; 2 TOBB-ETU Hospital, Clinic of Biochemistry and Clinical Biochemistry, Ankara, Turkey; 3 TOBB-ETU Hospital, Clinic of Pediatrics, Ankara, Turkey

**Keywords:** Thalassemia, Variant, Genetic counseling, Prenatal diagnosis, Beta globin gene, Turkish

## TO THE EDITOR,

Over 2000 gene variants were reported in the beta globin gene, including hemoglobin variants. These variants are important from clinical and genetic counseling points of view [1,2]. Recently a genetically related Turkish couple was referred to our department for genetic counseling for beta thalassemia carrier status. During premarital screening they were both diagnosed as beta thalassemia carriers by high pressure liquid chromatography analysis and whole blood count (Table 1). Genomic DNAs of both patients were extracted using the QIAamp DNA Blood Midi Kit (QIAGEN, Germany). The HBB gene was amplified using the following polymerase chain reaction (PCR) primers: forward (5’GCCAAGGACAGGTACGGCTG3’), reverse (5’CCCTTCCTATGACATGAACTTAACCAT3’) and forward (5’CAATGTATCATGCCTCTTTGCACC3’), reverse (5’GAGTCAAGGCTGAGGATGCGGA3’). Purifications were done using the ExoSAP purification program (Affymetrix Inc., USA). The BigDye Sequencing PCR technique was used for the analysis (Applied Biosystems, USA). Samples were analyzed with the SeqScape v2.5 analysis program. Common alpha globin gene deletions were analyzed according to the previously reported technique [3,4].

**Figure 1:**
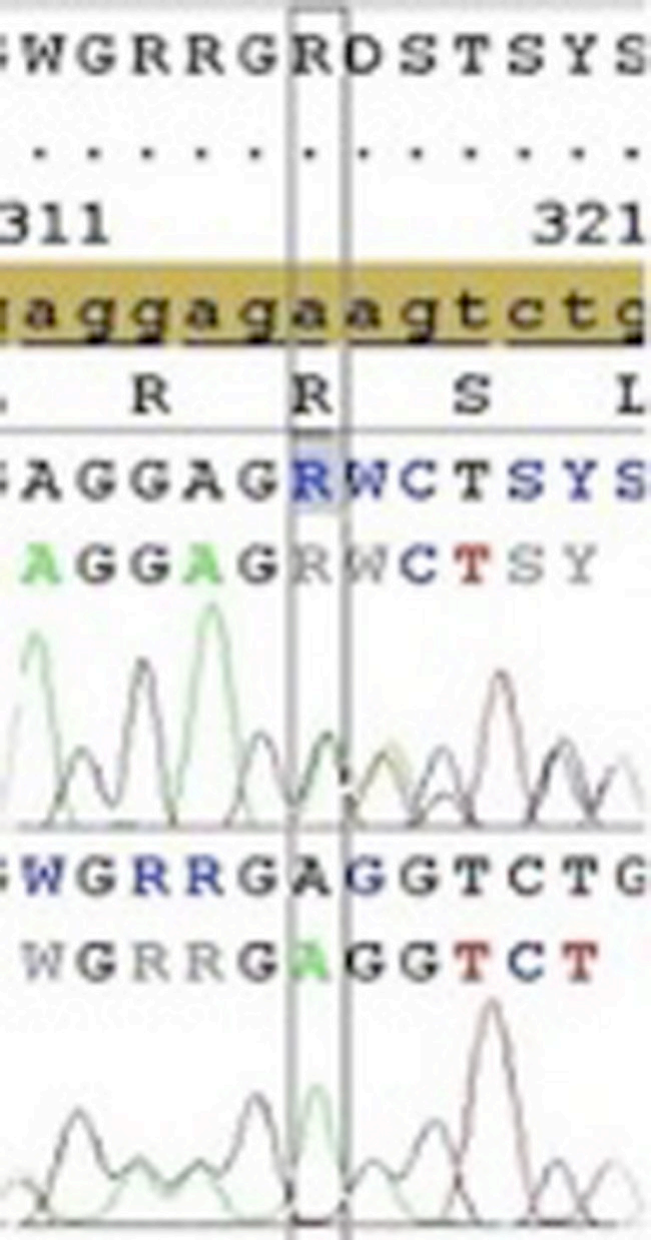
Sequencing data of the deletion at 25-26 AA (rs35497102).

**Figure 2:**
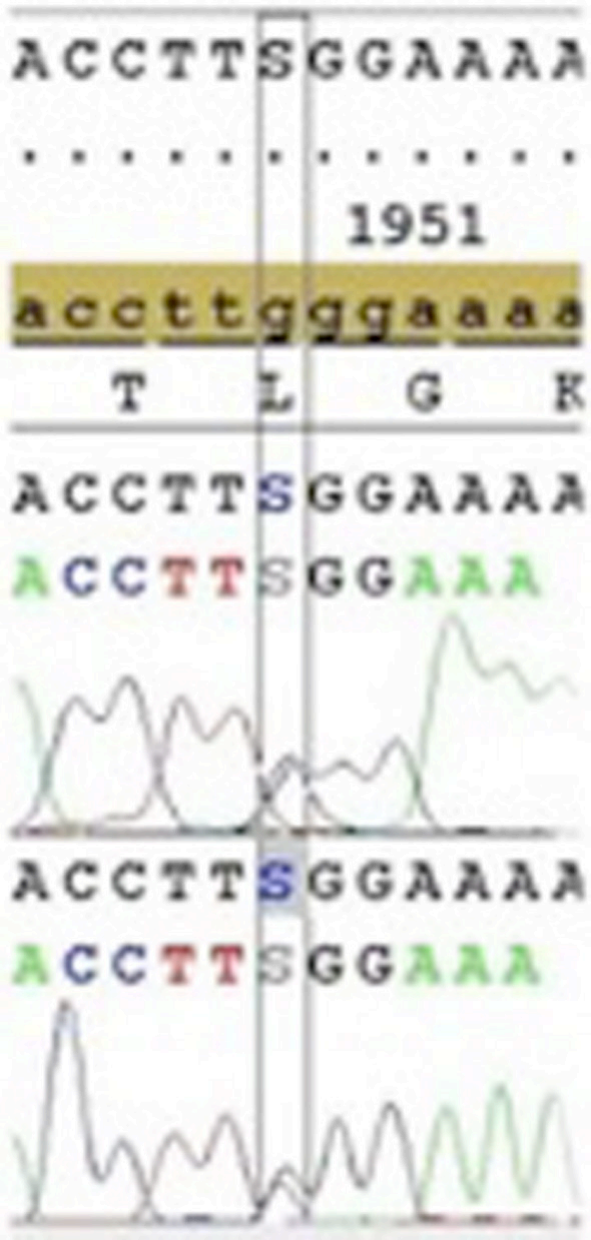
Single nucleotide polymorphism at 3’ UTR +101 G-C (+233 relative to termination codon).

Two different gene alterations were found in the beta globin gene of both partners (Table 1). One of them was a deletion at 25-26AA (rs35497102) (Figure 1). The other gene alteration was a single nucleotide polymorphism at 3’UTR+101 G-C +233 relative to the termination codon (rs12788013) (Figure 2). Neither of the individuals carried the common alpha thalassemia deletions.

Beta globin gene 3’UTR+101 G-C alteration is a single nucleotide polymorphism that was not previously classified and reported as a pathogenic variant [1,2]. It seems that carrying 3’UTR+101 G-C does not cause any additional clinical features in 25-26 del AA carriers. In this situation there is certainly more than one possibility to be mentioned in genetic counseling. 3’UTR+101 G-C being a single nucleotide variant resulting in a decreased expression of the gene causing the beta thalassemia major clinical picture is the most likely one. This is more evident when combined with a disease causing mutation, as previously reported by us and others [3,4,5,6,7]. However, from our family’s data, this is not valid, because they are beta thalassemia carriers. The main problem in this case is that an expression study was not performed for these individuals.

One of the possibilities for the inheritance pattern in this situation is that these two gene variants will be inherited in the “cis” position. In this case, from the genetic point of view, “in cis” is the only acceptable solution for the fetus, which will be similar to the parents. However, if it does not come in the “cis” position, there will be a possibility that the fetus may inherit 3’UTR+101 G-C in the homozygous state (from both parents). Unfortunately, not many publications have discussed similar conditions. For prenatal screening of the fetus, only the del 25-26 AA /3’UTR+101 G-C heterozygote state should be accepted as normal.

This case report highlights the need for investigating partnered beta thalassemia carriers by complete sequencing analysis of the beta globin gene if only one pathogenic mutation is detected by first-tier methods for the possibility of providing appropriate genetic counseling for couples at risk during prenatal genetic diagnosis.

## Figures and Tables

**Table 1:**
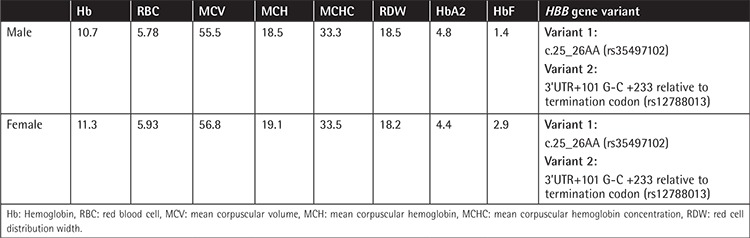
Whole blood count, hemoglobin A2 levels, and *HBB* gene mutation profile of the couple.
